# Retraction Note: The prevalence of electronic cigarettes vaping globally: a systematic review and meta-analysis

**DOI:** 10.1186/s13690-024-01345-x

**Published:** 2024-07-26

**Authors:** Hadi Tehrani, Abdolhalim Rajabi, Mousa Ghelichi- Ghojogh, Mahbobeh Nejatian, Alireza Jafari

**Affiliations:** 1 Social Determinants of Health Research Center, Mashhad University of Medical Sciences, Mashhad, Iran; 2 Department of Health Education and Health Promotion, School of Health, Mashhad University of Medical Sciences, Mashhad, Iran; 3 Environmental Health Research Center, Department of Biostatistics and Epidemiology, Faculty of Health, Golestan University of Medical Sciences, Gorgan, Iran; 4 Metabolic Disorders Research Center, Golestan University of Medical Sciences, Gorgan, Iran; 5 Social Determinants of Health Research Center, Gonabad University of Medical Sciences, Gonabad, Iran; 6 Department of Health Education and Health Promotion, School of Health, Social Development and Health Promotion Research Center, Gonabad University of Medical Sciences, Gonabad, Iran

**Retraction Note: Archives of Public Health (2022) 80:240**

10.1186/s13690-022-00998-w

The authors have retracted this article because it incorrectly reports the results of several studies included in their review. This impacts the overall results of their meta-analysis. The authors have been offered the opportunity to re-analyse their findings and to submit an updated version to the journal, which will be subjected to robust peer review.

All authors agree with this retraction.

